# Four small supernumerary marker chromosomes derived from chromosomes 6, 8, 11 and 12 in a patient with minimal clinical abnormalities: a case report

**DOI:** 10.1186/1752-1947-4-239

**Published:** 2010-08-03

**Authors:** Joaquín Fernández-Toral, Laura Rodríguez, Ana Plasencia, María Luisa Martínez-Frías, Elisabeth Ewers, Ahmed B Hamid, Monika Ziegler, Thomas Liehr

**Affiliations:** 1 Pediatría y jefe de sección de genética pediatrica del HUCA, Oviedo, Spain; 2 AbaCid-Genética Hospital de Madrid Norte Sanchinarro, Madrid, Spain; 3 Servicio de genética del HUCA. Oviedo, Spain; 4 Estudio Colaborativo Español de Malformaciones Congénitas (ECEMC) del Centro de Investigación sobre Anomalías Congénitas (CIAC), Instituto de Salud Carlos III, Ministerio de Sanidad y Consumo, Madrid, Spain; 5 Jena University Hospital, Institute of Human Genetics and Anthropology, Jena, Germany

## Abstract

**Introduction:**

Small supernumerary marker chromosomes are still a problem in cytogenetic diagnostic and genetic counseling. This holds especially true for the rare cases with multiple small supernumerary marker chromosomes. Most such cases are reported to be clinically severely affected due to the chromosomal imbalances induced by the presence of small supernumerary marker chromosomes. Here we report the first case of a patient having four different small supernumerary marker chromosomes which, apart from slight developmental retardation in youth and non-malignant hyperpigmentation, presented no other clinical signs.

**Case presentation:**

Our patient was a 30-year-old Caucasian man, delivered by caesarean section because of macrosomy. At birth he presented with bilateral cryptorchidism but no other birth defects. At age of around two years he showed psychomotor delay and a bilateral convergent strabismus. Later he had slight learning difficulties, with normal social behavior and now lives an independent life as an adult. Apart from hypogenitalism, he has multiple hyperpigmented nevi all over his body, short feet with pes cavus and claw toes. At age of 30 years, cytogenetic and molecular cytogenetic analysis revealed a karyotype of 50,XY,+min(6)(:p11.1-> q11.1:),+min(8)(:p11.1->q11.1:),+min(11)(:p11.11->q11:),+min(12)(:p11.2~12->q10:), leading overall to a small partial trisomy in 12p11.1~12.1.

**Conclusions:**

Including this case, four single case reports are available in the literature with a karyotype 50,XN,+4mar. For prenatally detected multiple small supernumerary marker chromosomes in particular we learn from this case that such a cytogenetic condition may be correlated with a positive clinical outcome.

## Introduction

Multiple small supernumerary marker chromosomes (sSMC) with diverse sSMC derived from different chromosomal origin are rarely reported. According to Liehr [1], up to now 46 such cases were reported: 33 cases with two different sSMC, four cases each with three or four different sSMC, two each with six and seven sSMC, and one case with five sSMC. Overall, only seven of the 46 cases (= 15%) were reported as without clinical signs (according to Liehr [1] cases 2-14, 2-17, 2-23, 2-26, 2-29, 3-3 and 7-1).

Patients with multiple sSMC constitute a sub-group of patients with sSMC [2,3]. Little is known about the formation of sSMC in general [1-3] or about multiple sSMC specifically [4]. As reported previously, chromosomes 6, 3, 5, X, 1, 7, and 12 are over-represented in multiple sSMC compared to their contribution to single sSMC [4].

Here we report the first case with four sSMC derived from chromosomes 6, 8, 11 and 12, with almost no clinical signs.

## Case presentation

Our patient was a 30-year-old Spanish Caucasian man; the third child from healthy and non-consanguineous parents. The first child was a healthy boy and the second child was also a boy who died after two days due to hyaline membrane disease and prematurity. Our patient was delivered by caesarean section after 39 gestational weeks because of macrosomy, with a weight of 4250 g and an Apgar score of three, thus, intensive reanimation was required. Within five hours of life he suffered apnea. He was also hypoglycemic and hypocalcemic, but responded well to treatment without suffering a recurrence. Clinical examination showed bilateral cryptorchidism. During her pregnancy our patient's mother was treated with diazepam towards the end of the pregnancy.

When our patient was 19 months old, his weight and length were two standard deviations below normal. During further development, he showed psychomotor delay and a bilateral convergent strabismus; also he started walking when he was 22 months old. At the age of 10 years, his testes were surgically descended. And at the age of 13 years the strabismus was corrected. At school he had slight learning difficulties, with normal social behavior. He later left studying to become a painter.

**Figure 1:**
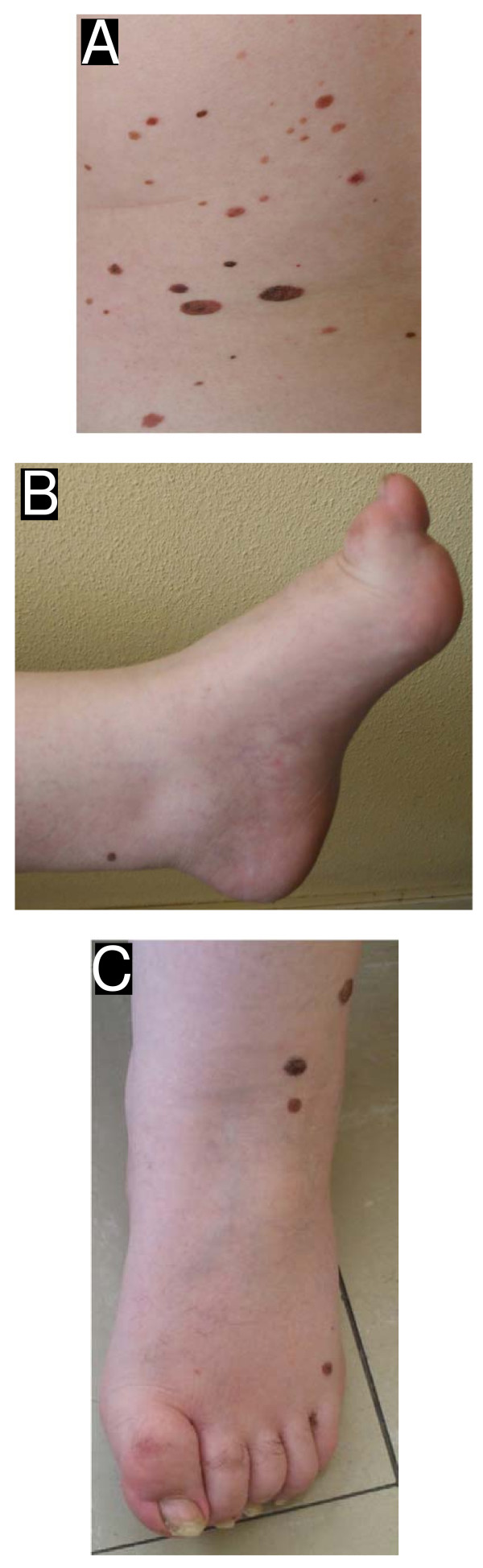
**View of the patient at age of 30 years**. (A) Multiple hyperpigmented nevi at the trunk. (B,C) Multiple hyperpigmented nevi at the foot which was too short, showed a pes cavus and claw toes.

When he was 22 years old, he had no facial dysmorphism, he weighed 89 kg, his height was 165 cm and he had a corporal index mass of 32.7. He had hypogenitalism, with a short thick penis (6 cm), and testes of 8 and 10 cc. He has multiple hyperpigmented nevi all over his body, showing no sign of malignancy after biopsy (Figure 1A,C). He also had a left vesicoureteral reflux grade III, with normal renal function. His cardiac, audition and fundus of the eye examinations were normal, as was his blood biochemistry. His feet are short with a pes cavus and claw toes (Figure 1B,C). At this time, he was referred to a Genetic Laboratory and one sSMC was found in his karyotype, which was considered to be *de novo *because his parents had normal karyotypes. Now, at the age of 30 years a new blood sample for cytogenetic analysis was requested. Surprisingly, the high resolution G-band karyotype attained from this sample showed the presence of a relatively big SMC, together with the presence of three additional tiny SMCs in most cells. This cytogenetic analysis revealed a karyotype of 50,XY,+mar1,+mar2,+mar3,+mar4.

**Figure 2:**
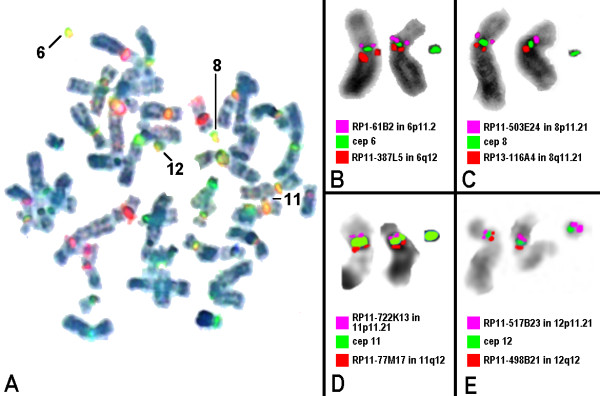
**FISH results obtained on the chromosomes of the reported patient**. (A) cenM-FISH revealed that the four sSMC were derivatives of chromosomes 6, 8, 11, and 12. (B-E) subcenM-FISH revealed absence of euchromatic material in sSMC derived from chromosomes 6, 8 and 11 and presence of centromere near material on the sSMC(12).

To further characterize the sSMC centromere-specific multicolor fluorescence *in situ *hybridization (cenM-FISH [5]) was carried out. From this the chromosomal origin of the sSMC was determined as 6, 8, 11 and 12 (Figure 2A). By sub-centromere specific M-FISH (subcenM-FISH [6,7]) (Figure 2B-E) it was shown that the sSMC derived from chromosomes 6, 8 and 11 do not contain any detectable euchromatic material. Only for the derivative of chromosome 12 centromere-near material in 12p12.1 could be detected. The final karyotype was 50,XY,+min(6)(:p11.1->q11.1:),+min(8)(:p11.1->q11.1:),+min(11)(:p11.11->q11:),+min(12)(:p11.2~12->q10:).

## Discussion

Here we report the fourth unusual case with four different sSMC and the 34th case with multiple sSMC. It is the eighth case with no or only minor clinical signs due to the sSMC presence. The only detectable sSMC-related chromosomal imbalance is a small partial trisomy 12p11.2~12.1. According to Liehr [8] there are several cases with a partial trisomy 12p12 due to an sSMC which were all clinically normal. Thus, this region seems to be a potentially transmittable unbalanced chromosomal abnormality (UBCA) without causing clinical problems (see case 12-O-p11.1/1-1 [8]). Similar UBCA were recently reported for a multitude of chromosomal regions [9] and especially for the centromere near regions [3]. Thus, it is not clear if the sSMC have a positive correlation with the observed clinical symptoms.

Moreover, it is interesting that the multiple sSMC derive in the present case from chromosomes 6, 8, 11 and 12. Chromosomes 6 and 12 are over-represented in multiple sSMC cases reported to date compared to their contribution to single sSMC [4]. This might point towards a specific way of formation of multiple sSMC during meiosis [10].

## Conclusions

The present case confirms that multiple sSMC may be correlated with an almost normal clinical outcome. This is especially important for the correct genetic counseling of similar pre-natal cases. Furthermore, a small partial trisomy

12p11.2~12.1 seems to correlate largely to no clinical effects. Finally, involvement of chromosome 6 in sSMC formation seems to be correlated with the tendency of multiple sSMC formation.

## Competing interests

The authors declare that they have no competing interests.

## Authors' contributions

LR performed the cytogenetic studies in the present case. JFT and AP collected the data relative to this case report and provided genetic counseling to the parents. MLMF supervised the cytogenetic analysis as Director of the ECEMC. EE, ABH, MZ and TL did the molecular cytogenetic analysis and interpretation. TL drafted the paper and all authors contributed to the finalizing of the manuscript.

## Consent

Written informed consent was obtained from the patient for publication of this case report and any accompanying images. A copy of the written consent is available for review by the Editor-in-Chief of this journal.
